# Fault Diagnostics Based on the Analysis of Probability Distributions Estimated Using a Particle Filter

**DOI:** 10.3390/s24030719

**Published:** 2024-01-23

**Authors:** András Darányi, János Abonyi

**Affiliations:** HUN-REN-PE Complex Systems Monitoring Research Group, Department of Process Engineering, University of Pannonia, Egyetem u. 10, P.O. Box 158, H-8200 Veszprem, Hungary; daranyi.andras@mk.uni-pannon.hu

**Keywords:** fault diagnostics, particle filter, monitoring, sensor fault, actuator fault

## Abstract

This paper proposes a monitoring procedure based on characterizing state probability distributions estimated using particle filters. The work highlights what types of information can be obtained during state estimation and how the revealed information helps to solve fault diagnosis tasks. If a failure is present in the system, the output predicted by the model is inconsistent with the actual output, which affects the operation of the estimator. The heterogeneity of the probability distribution of states increases, and a large proportion of the particles lose their information content. The correlation structure of the posterior probability density can also be altered by failures. The proposed method uses various indicators that characterize the heterogeneity and correlation structure of the state distribution, as well as the consistency between model predictions and observed behavior, to identify the effects of failures.The applicability of the utilized measures is demonstrated through a dynamic vehicle model, where actuator and sensor failure scenarios are investigated.

## 1. Introduction

The objective of this study was to investigate the information that can be obtained through state estimation using a particle filter (PF) [1] and how it can aid in fault diagnosis tasks. The aim was to develop and systematize methods that are suitable for diagnosing and visualizing the operation of particle filters (PF). Gaining insight into the operation of the filter can reveal significant features for fault diagnosis.

Fault diagnosis is responsible for detecting and identifying faults and taking corrective action. Different diagnostic approaches are used to detect and identify faults. The two main approaches to quantitative fault diagnostics are data-driven [2] and model-based [3]. In the former case, machine learning models are trained on historical data about the operation. If labeled data for the operation under faulty conditions are available, supervised machine learning can be used to classify data into different operation modes. There are several appropriate methods for this classification, including neural networks, support vector machines [4], principal component analysis [5], and decision trees [6]. The effectiveness of fault-type differentiation methods is heavily influenced by the features used. Therefore, sophisticated feature engineering procedures are typically required [7]. In practical cases, the lack of data about the faulty mode means that the normal mode data are over-represented [8], which can be problematic in supervised methods. In this case, the goal is to identify data that differ from the data generated under normal conditions, which is a classic anomaly-detection task [9]. Detection can be based on the absolute value of a characteristic or its trend [10]. A change in the statistical characteristics, such as variance, of the relevant feature in a fixed-length sliding window may also indicate an anomaly [11]. In addition to the use of appropriate features, the optimal choice of thresholds that distinguish the abnormal domain from the normal domain is critical in anomaly detection [12]. The thresholds should be chosen to provide the required sensitivity to faults, while keeping the false alarm rate as low as possible, even under dynamic conditions [13]. Several methods are available to select an appropriate threshold in the presence of labeled erroneous data [14]. Binary classification metrics can be used to fine-tune the threshold(s) [15]. In a simple case, the selection is made heuristically based on expert knowledge. Under more dynamic conditions, adaptive thresholds are proposed instead of fixed values, to cope with the difficulties caused by inference [16]. There are advanced methods beyond threshold-based approaches for anomaly detection, which use statistical hypothesis testing [17] or Bayesian inference [18].

The essence of model-based fault diagnostics systems is to compare the predicted behavior based on the system model with the actual observed behavior [19]. The deviations between model prediction and observation, called residuals, represent the potential faults of the system. They are calculated on the basis of analytical redundancy, which involves the algebraic and temporal relationships between the system’s states, inputs, and outputs [20]. This approach requires precise and accurate predictions. Prediction error metrics can be used to assess the performance of estimation algorithms [21]. The classic Kalman filter is a widely used technique for model-based fault diagnosis [22]. However, it is limited to linear systems. In non-linear dynamics, the extended Kalman filter [23] and the unscented Kalman filter [24] can be useful, but these require linearization and the assumption of Gaussian noise. PFs offer more powerful estimations in non-linear and non-Gaussian systems [25], which are prevalent in many real-world applications. PFs are based on Monte Carlo sampling, which allows them to approximate complex probability distributions. A PF is suitable for tracking the state of hybrid systems, making it applicable to systems with varying dynamics [26].

PF-based fault detection employs two approaches. One is the comparison of residuals with threshold values. This approach was applied in the diagnosis of faults in electric aircraft systems [27] and in induction generators used in wind turbines [28]. The other approach is based on the likelihood of particles. The logarithm of the likelihood of the output particles considering a measurement density is summed, and that value is examined in a sliding window to avoid false alarms due to disturbances.This value is then evaluated using a threshold similar to the previous one. Moving average [29], exponential smoothing [30], or simple summation in a window [31] are generally used to form a decision function from the sum of particle likelihoods.

Fault isolation, however, is a more complex task that requires more sophisticated approaches than a basic state estimation. A common approach in PF-based fault diagnosis is to track changes in the parameters [32,33]. In this method, the underlying assumption is that faults in a system can be represented as changes in the system’s parameters. By monitoring and estimating these parameter changes, faults can be detected and diagnosed. By comparing the estimated parameters with their expected values, any deviations can be identified as potential faults. This approach can be useful, for example, during tool wear diagnostics [34]. Another approach is to use a hybrid system model with discrete modes [35,36]. In this method, the state space is extended to include additional discrete variables that represent the different modes or states of the system faults. Each particle in the filter carries not only the state estimate but also the mode estimate. Each mode has different dynamics. The fault identification process is based on the estimation of continuous states and the calculation of the most probable discrete state [26,37,38]. This approach is particularly useful when the system has multiple possible fault modes that can occur simultaneously or sequentially. This allows for more accurate fault detection and identification through explicitly modeling the different fault scenarios. Furthermore, particle filter-based fault diagnostic methods can use dedicated observers [39]. Dedicated observers are specialized models designed to capture specific fault signatures or symptoms. These observers usually specialize in a particular sensor, whose signal is not taken into account when estimating the state. In the event of the failure of a particular sensor, the estimate of the dedicated observer remains unaffected, while the others are distorted [40]. This phenomenon can be detected through hypothesis testing [25], using the likelihood of a particular observer [40] or using the likelihood ratio of a particular observer to a main observer [41]. The development and application of fault isolation algorithms require detailed knowledge of a fault model. These fault-specific models and knowledge are not always available, so these tools cannot be easily applied for the identification of novel faults.

This study aims to investigate whether the analysis of state distributions estimated using a particle filter can provide valuable features for reliable fault detection and to isolate individual failure effects. Various indicators and visualizations are utilized to analyze the structure of the distribution that represents the state and its uncertainty, along with the operation of the estimation algorithm used to identify fault-specific information patterns. During the presented procedure, we examine three aspects: the homogeneity of the discrete distributions produced, the correlation structure between the state variables, and the update step for the filter.

The key contributions of this study can be summarized as follows:

- We provide a set of indicators to evaluate the probability distribution of the states estimated using the particle filter.
- This study investigates the homogeneity of probability distributions generated with a particle filter using probabilistic and information-theoretic metrics.
- The evolution of the correlation structure of estimated distributions over time is monitored.
- The consistency between model-predicted distributions and measurements is monitored.
- The proposed indicators are demonstrated through a vehicle dynamics example.
- The effectiveness of the proposed metrics is examined using sensor and actuator failure scenarios.

The article is structured as follows: In Section 2, the methodology is introduced. Section 2.1 explains the particle filter state estimation algorithm. Section 2.2 describes the measures applied for the evaluation of the heterogeneity of the state-probability distributions estimated using a particle filter (Section 2.2.1), for the correlation structure of the states (Section 2.2.2), and for the consistency between the model predictions and observations (Section 2.2.3). Section 3 presents the application example within whose framework the analysis was carried out. Section 3.1 describes the model used. Section 3.2 explains the simulated scenarios. Section 3.3 describes the applied estimator parameters. Section 3.4 presents and discusses the results of the investigations. Finally, some concluding remarks are made.

## 2. Monitoring the Operation of the Particle Filter and Its Estimated Distributions

In this section, we first detail the particle filter-based state estimation. The proposed monitoring procedure is then explained, which involves investigating three aspects of the operation of the estimator:

- the heterogeneity of the estimated state distributions is examined over time;
- the correlation pattern between the state variables is monitored;
- the consistency between model predictions and measurements is also qualified.

The metrics used for these three investigations are presented.

### 2.1. State Estimation with Particle Filter

Bayesian state estimation aims to recursively calculate the posterior probability density function of the state using an assumption about the evolution of the $d_{x}$-dimensional state $x^{\left(t\right)}$ and the $d_{y}$-dimensional measurement $y^{\left(t\right)}$ in each time instant *t*. The general discrete state-space model describes how the state evolves in time:

(1) $$x^{\left(t\right)}=f\left(x^{\left(t-1\right)},u^{\left(t-1\right)},v^{\left(t-1\right)}\right),\;v^{\left(t-1\right)}∼N\left(0,Q\right)$$

where *f* is the state-transition function, $u^{\left(t-1\right)}$ stands for the input vector, and $v^{\left(t-1\right)}$ denotes the process noise vector in the (t−1)th time instant. The process noise is assumed to follow a zero-mean Gaussian probability density function (PDF) with a covariance matrix Q with $d_{x}\times d_{x}$ dimensions. The measurement equation creates a link between the information about the state and the noisy measurements:

(2) $$y^{\left(t\right)}=h\left(x^{\left(t\right)},w^{\left(t\right)}\right)\;w^{\left(t\right)}∼N\left(0,R\right)$$

where *h* is the measurement function, $y^{\left(t\right)}$ is the measurement in time *t*, and $w^{\left(t\right)}$ represents the measurement noise. This noise is also assumed to be a zero-mean Gaussian PDF with covariance matrix R with dimensions $d_{y}\times d_{y}$.

The particle filter [1] applies discrete sampling densities generated using Monte Carlo simulations to estimate the internal states $x^{\left(t\right)}$ of a dynamics system. In this method, the posterior density of the state is approximated in each *t*th discrete time instant using a set of $N_{s}$ random samples called particles represented by $\left\{x_{i}^{\left(0:t\right)},i=1,\ldots ,N_{s}\right\}$ support points and $\left\{w_{i}^{\left(0:t\right)},i=1,\ldots ,N_{s}\right\}$ associated weights. This discrete sampling density is expressed as

(3) $$p\left(x^{\left(0:t\right)}|y^{\left(1:t\right)}\right)\approx \sum_{i=1}^{N_{s}}w_{i}^{\left(t\right)}\delta \left(x^{\left(0:t\right)}-x_{i}^{\left(0:t\right)}\right)$$

where δ is the Dirac delta function. The associated weights $w_{i}^{\left(t\right)}$ are obtained based on the importance sampling technique [42]:

(4) $$w_{i}^{\left(t\right)}\propto \frac{p(x_{i}^{\left(0:t\right)}|y^{\left(1:t\right)})}{q(x_{i}^{\left(0:t\right)}|y^{\left(1:t\right)})}$$

where *q* denotes the importance density function defined as:

(5) $$q\left(x^{\left(0:t\right)}|y^{\left(1:t\right)}\right)=q\left(x^{\left(t\right)}|x^{\left(0:t-1\right)},y^{\left(1:t\right)}\right)q\left(x^{\left(0:t-1\right)}|y^{\left(1:t-1\right)}\right)$$

To derive the appropriate weights, we need to express the joint posterior density as follows:

(6) $$\begin{array}{c} p\left(x^{\left(0:t\right)}|y^{\left(1:t\right)}\right)=\frac{p\left(y^{\left(t\right)}|x^{\left(0:t\right)},y^{\left(1:t-1\right)}\right)p\left(x^{\left(0:t\right)}|y^{\left(1:t-1\right)}\right)}{p(y^{\left(t\right)}|y^{\left(1:t-1\right)})}\\ =\frac{p\left(y^{\left(t\right)}|x^{\left(t\right)}\right)p\left(x^{\left(t\right)}|x^{\left(t-1\right)}\right)}{p(y^{\left(t\right)}|y^{\left(1:t-1\right)})}p\left(x^{\left(0:t-1\right)}|y^{\left(1:t-1\right)}\right)\\ \propto \;p\left(y^{\left(t\right)}|x^{\left(t\right)}\right)p\left(x^{\left(t\right)}|x^{\left(t-1\right)}\right)p\left(x^{\left(0:t-1\right)}|y^{\left(1:t-1\right)}\right) \end{array}$$

By substituting Equations (5) and (6) into Equation (4), the weight update equation can be written as follows:

(7) $$\begin{array}{c} {\tilde{w}}_{i}^{\left(t\right)}\propto \frac{p\left(y^{\left(t\right)}|x_{i}^{\left(t\right)}\right)p\left(x_{i}^{\left(t\right)}|x_{i}^{\left(t-1\right)}\right)p\left(x_{i}^{\left(0:t-1\right)}|y^{\left(1:t-1\right)}\right)}{q\left(x_{i}^{\left(t\right)}|x_{i}^{\left(0:t-1\right)},y^{\left(1:t\right)}\right)q\left(x_{i}^{\left(0:t-1\right)}|y^{\left(1:t-1\right)}\right)}\\ =\frac{p\left(y^{\left(t\right)}|x_{i}^{\left(t\right)}\right)p\left(x_{i}^{\left(t\right)}|x_{i}^{\left(t-1\right)}\right)}{q(x_{i}^{\left(t\right)}|x_{i}^{\left(0:t-1\right)},y^{\left(1:t\right)})}w_{i}^{\left(t-1\right)} \end{array}$$

The tilde superscript (${\tilde{w}}_{i}^{t}$) indicates that the posterior normalization factor is not taken into account here (Equation (6)).

Due to the Markov property, $q\left(x^{\left(t\right)}|x^{\left(0:t-1\right)},y^{\left(1:t\right)}\right)=q\left(x^{\left(t\right)}|x^{\left(t-1\right)},y^{\left(t\right)}\right)$.

The choice of importance density is crucial in designing a particle filter. The most commonly applied sub-optimal choice is the state transition function:

(8) $$q\left(x^{\left(t\right)}|x_{i}^{\left(t-1\right)},y^{\left(t\right)}\right)=p\left(x^{\left(t\right)}|x_{i}^{\left(t-1\right)}\right)$$

By substituting Equation (8) into Equation (7), we can express the weight update formula as

(9) $${\tilde{w}}_{i}^{\left(t\right)}\propto w_{i}^{\left(t-1\right)}p\left(y^{\left(t\right)}|x_{i}^{\left(t\right)}\right)$$

It is important to note that the normalization factor of the posterior density (Equation (6)) is still unknown. Therefore, we have to normalize the importance of weights:

(10) $$w_{i}^{\left(t\right)}=\frac{{\tilde{w}}_{i}^{\left(t\right)}}{\sum_{j=1}^{N_{s}}{\tilde{w}}_{j}^{\left(t\right)}}$$

Then, the posterior density of *t* time instant can be approximated as

(11) $$p\left(x^{\left(t\right)}|y^{\left(t\right)}\right)\approx \sum_{i=1}^{N_{s}}w_{i}^{\left(t\right)}\delta \left(x^{\left(t\right)}-x_{i}^{\left(t\right)}\right)$$

A well-known challenge of the weight update formula is the sample degeneracy problem [43]. As the number of iterations increases, a small fraction of particles receive larger and larger weights, while the other particle weights become negligible. Thus, after a while, only one particle represents the state. In this case, the uncertainty of the state is not represented. Consequently, the algorithm cannot track the changes in the process. The extent of degeneracy is monitored by approximating the effective sample size in each iteration [44]. The effective sample size can be approximated as

(12) $${\hat{N}}_{eff}=\frac{1}{\sum_{i=1}^{N_{s}}{\left(w_{i}^{\left(t\right)}\right)}^{2}}$$

To avoid the undesirable phenom of sample degeneracy, the particles are resampled when the effective sample size ${\hat{N}}_{eff}$ falls below a predetermined threshold value [45]. During the resampling process, new particles are randomly selected from the set of existing particles ${\left\{x_{i}^{\left(t\right)}\right\}}_{i=1}^{N_{s}}$ with replacement. The probability of selecting $x_{i}^{\left(t\right)}$ is proportional to its weight $w_{i}^{\left(t\right)}$. All weights are reset to $1/N_{s}$ after resampling. Resampling and reweighting allow keeping the shape of the posterior distribution, so that all particles contribute equally to the information content.

The following pseudocode briefly summarizes the particle filter algorithm (Algorithm 1):
**Algorithm 1 The particle filter algorithm****Input:** A set of measurements (mostly in real-time defined as streaming data, but the whole time series may be already available) $y^{\left(0:t\right)}$, a set of control inputs (as streaming data or whole time series) $u^{\left(0:t\right)}$, the model is defined by the *f* and *h* functions, and the parameters of the algorithm $\left(R,Q,N_{s},\epsilon \right)$
**Output:** Set of state samples $x_{i}^{\left(t\right)}$ and the associated weights $w_{i}^{\left(t\right)}$
  1:**function** Particle filtering($y_{i}^{\left(0:t\right)},u^{\left(0:t\right)},f,h,R,Q,N_{s},\epsilon$)  2:    **Step 1: Initialization of particles**  3:    **for** $i=1,\ldots ,N_{s}$ **do**  4:          Draw samples $x_{i}^{\left(t=0\right)}$ from the priori distribution of state.  5:          Initialize their weights $w_{i}^{\left(t=0\right)}=1/N_{s}$.  6:    **end for**  7:    **Step 2: Prediction**  8:    **for** $i=1,\ldots ,N_{s}$ **do**  9:          Propagate particles $x_{i}^{\left(t\right)}$ with state-transition model *f* (Equation (1)).10:    **end for**11:    **Step 3: Update weights**12:    **for** $i=1,\ldots ,N_{s}$ **do**13:          Calculate the likelihood of each prior particle $p(y^{\left(t\right)}|x_{i}^{\left(t\right)})$ based on the new measurement $y^{\left(t\right)}$ and measurement equation *h* (Equation (2)).14:          Incorporate the information from the new measurement $y^{\left(t\right)}$ by updating the particle weights $w_{i}^{\left(t\right)}$ according to Equations (7) and (10).15:    **end for**16:    **Step 4: Resampling**17:    Calculate the effective sample size ${\hat{N}}_{eff}$ according to Equation (12).18:    **if** ${\hat{N}}_{eff}<\epsilon$ **then**19:          Resample particles $x_{i}^{\left(t\right)}$ and reset their weights $w_{i}^{\left(t\right)}$ to $1/N_{s}$.20:    **end if**21:    **Repeat**22:    **for** t=1,…,∞ **do**23:          Repeat Steps 2–4.24:    **end for**25:**end function**


The computational effort of the algorithm is mainly related to the complexity of the model used, since the simulation based on the model is carried out in an amount of time corresponding to the number of particles.

### 2.2. Monitoring the Behavior of a Particle Filter

Failure will cause the model-estimated value to be inconsistent with the observed value, increasing the uncertainty. This uncertainty is reflected in a decrease in the probability weights of the state samples [46]. Lower weights result in a higher resampling frequency, decreasing the homogeneity of the state distribution. Furthermore, it is assumed that the presence of a defect changes the correlation pattern in the state particle distribution, which can be detected. In this study, different monitoring techniques are applied to track the operation of the estimator in case of different types of faults. Probabilistic and information-theoretic measures are used to evaluate the homogeneity of the posterior distributions. The correlation structure of the estimated state particles in the distributions and their time evolution are investigated. In the filter update step, the information content of the measurement is incorporated into the assumptions made about the model, which, if consistent, will yield a more informative posterior compared to the predicted distribution. The information surplus between the posterior and prior distributions, the similarity of the eigenvectors of their covariance matrix, and the distance between the prior distributions and the distributions representing the measurement are measured to test this.

#### 2.2.1. Evaluation of the Compactness and Heterogeneity of the Posterior Distribution

The uncertainty and reliability of the posterior carry important information for us. The appearance of a fault involves an inconsistency between the model and the measurement. As a result, the homogeneity of the posterior distribution and the diversity of the state particles decreases, and their estimated probability also decreases. In contrast, during the normal operation mode, when the estimation is consistent, the estimated state values are evenly distributed around the true state, and most of them carry significant probability weights. This implies high confidence in the estimates. Four indicators will be used to monitor these phenomena.

The theoretical definition of effective sample size $N_{eff}$ corresponds to the number of independent samples that, if generated directly from the target distribution, would result in an estimation efficiency equivalent to that achieved using the MCMC or IS algorithms [44]. The calculation is performed using the widely accepted approximation formula (Equation (12)).

The logarithmic likelihood function provides a good indicator of the probability that the posterior distribution represents the actual state. Its value gives an indication of how closely the prior density coincides with the measurement density. Given $N_{s}$ number of state particles $x_{i}^{\left(t\right)}$ in a discrete predictive distribution $P_{prior}\left(x^{\left(t\right)}\right)$ at the *t*th time instant, the logarithmic likelihood function is expressed using the sum of the logarithm of likelihoods of the particles conditioned on measurement $y^{\left(t\right)}$:

(13) $$\ell \left(P_{prior}\left(x^{\left(t\right)}\right)\right)=\sum_{i=1}^{N_{s}}\log\left(p\left(y^{\left(t\right)}|x_{i}^{\left(t\right)}\right)\right)$$

Shannon entropy [47] is a measure suitable for quantifying the uncertainty and heterogeneity of a distribution. Given $N_{s}$ number of particles $x_{i}^{\left(t\right)}$ and their estimated probability densities $p(x_{i}^{\left(t\right)}|y^{\left(t\right)})$ in a discrete posterior distribution $P_{post}\left(x^{\left(t\right)}\right)$ at *t*th time instant, the entropy formula is expressed as follows:

(14) $$H\left(P_{post}\left(x^{\left(t\right)}\right)\right)=-\sum_{i=1}^{N_{s}}p\left(x_{i}^{\left(t\right)}|y^{\left(t\right)}\right)\log_{2}p\left(x_{i}^{\left(t\right)}|y^{\left(t\right)}\right)=-\sum_{i=1}^{N_{s}}w_{i}^{\left(t\right)}\log_{2}w_{i}^{\left(t\right)}$$

The entropy calculated from the weights is used to infer the heterogeneity of the posterior. If it is high, then the particles are identical; we do not know which corresponds better to the actual state. If the weights are concentrated on a few particles, it takes a low value.

Another measure of the diversity of particles in terms of their information content is the population diversity factor [48]. The population diversity factor is described by the following formula:

(15) $$D_{pop}=s\left(w_{max}^{\left(t\right)}-w_{av}^{\left(t\right)}\right)/w_{av}^{\left(t\right)}$$

where $w_{max}^{\left(t\right)}$ denotes the maximum weight, $w_{av}^{\left(t\right)}$ denotes average of the upper 50% of weights, and *s* stands for a scale factor that generally takes a value between 3 and 6.

#### 2.2.2. Investigating Correlations between the State Variables of Particles

When a fault occurs, it can lead to increased uncertainty and inconsistency in state estimates. This increased uncertainty is reflected in the covariance matrix of the state estimates. A sudden change in the covariance of certain state variables may indicate the presence of a fault that affects those variables. Depending on the type of fault, the covariance structure of the state estimates may change over time. Tracking these changes can help diagnose the fault and understand its evolution. In the presence of a fault, certain state variables can become more correlated than they were in the fault-free condition. The eigenvectors of the prior and posterior distributions provide insight into the correlation structure of data. They describe the directions or axes of maximum variance in the data space. The direction of each eigenvector represents a particular correlation pattern between the variables. Eigenvectors associated with high eigenvalues capture strong correlations, while those with low eigenvalues represent weak or negligible correlations. If the eigenvectors remain consistent or exhibit minimal changes, this suggests that the dominant directions of variation in the data remain relatively stable. However, significant changes in the eigenvectors indicate variations in the underlying structure or dynamics of the data. Changes in the eigenvectors can indicate mode switching or transitions between different patterns or clusters in the data, which may be caused by a failure. The eigenvectors and eigenvalues are used to track the covariance structure of the posterior distributions at each *t* time instant, allowing the capture of changes: from each $d_{x}\times d_{x}$ dimensional covariance matrices $C^{\left(t\right)}$ of posterior distribution $P_{post}\left(x_{i}^{\left(t\right)}\right)$, the eigenvectors $V=[v_{1}^{\left(t\right)},v_{2}^{\left(t\right)},\ldots ,v_{d_{x}}^{\left(t\right)}]$ and corresponding eigenvalues $\lambda_{1}^{\left(t\right)},\lambda_{2}^{\left(t\right)},\ldots ,\lambda_{d_{x}}^{\left(t\right)}$ are calculated.

Note that particle weights $\left\{w_{i}^{\left(t\right)}\right\}$ are taken into account when calculating the covariance of a posterior distribution $P_{post}\left(x_{i}^{\left(t\right)}\right)$.

#### 2.2.3. Investigation of the Weight Update Process to Infer the Consistency between the Model and Observations

During the update step, the information content of a measurement is incorporated into the posterior distribution. Each particle is weighted according to the likelihood it has in the probability distribution, representing the uncertainty of the measurement. If the model and measurement are consistent, the two distributions overlap to a large extent and a large proportion of particles contribute to the posterior information content. In the case of a fault, however, the two distributions are further apart, so the degree of overlap is smaller. Then, the posterior distribution will be skewed, and the particle weights will be concentrated at the edge of the distribution. This will result in a more heterogeneous posterior, which is more ordered in terms of information content. The Kullback–Leibler (KL) divergence [49] is a suitable measure of the divergence between the information content of the prior and posterior in fault diagnosis [36]. It measures relative entropy; that is, how much extra information is needed before specifying the value of the state variable x as a result of using the prior instead of the posterior distribution. Let $P_{prior}\left(x^{\left(t\right)}\right)$ and $P_{post}\left(x^{\left(t\right)}\right)$ be prior and posterior distributions of the *t*th time instant. The KL divergence can be expressed for them as

(16) $$D_{KL}(P_{prior}\left(x_{i}^{\left(t\right)}\right)\left|\right|P_{post}\left(x_{i}^{\left(t\right)}\right))=\sum_{i=1}^{N_{s}}p\left(x^{\left(t\right)}|x_{i}^{\left(t-1\right)}\right)ln\frac{p(x^{\left(t\right)}|x_{i}^{\left(t-1\right)})}{p(x_{i}^{\left(t\right)}|y^{\left(t\right)})}$$

The degree of overlap between the prior and the distribution characterizing the uncertainty of the measurement is quantified using the Bhattacharyya distance [49]. The Bhattacharyya distance between the predictive and measurement distributions $P_{prior}\left(x^{\left(t\right)}\right)$ and $P_{meas}\left(x^{\left(t\right)}\right)$ can be calculated based on their densities $p(x^{\left(t\right)}|x_{i}^{\left(t-1\right)})$ and $p(y^{\left(t\right)}|x_{i}^{\left(t-1\right)})$:

(17) $$D_{B}(P_{prior}\left(x^{\left(t\right)}\right)\left|\right|P_{meas}\left(x^{\left(t\right)}\right))=-ln\left(\sum_{i=1}^{N_{s}}\sqrt{p\left(x^{\left(t\right)}|x_{i}^{\left(t-1\right)}\right)p\left(y^{\left(t\right)}|x_{i}^{\left(t-1\right)}\right)}\right)$$

If the measurement and the expected value are in sync, the update does not significantly change the correlation structure of the particles. In this case, the state dimensions with the highest variance point are roughly in the same direction in the prior and posterior distributions. The difference between the eigenvectors of each covariance matrix is examined on the basis of Krzanowskii similarity [50]. This measure indicates the similarity or alignment between the dimensions of the hyperplanes defined by the longest eigenvectors. Let us assume A and B are covariance matrices of $P_{prior}\left(x_{n}^{\left(t\right)}\right)$ and $P_{post}\left(x_{n}^{\left(t\right)}\right)$ prior and posterior distributions. The Krzanowsky similarity between A and B in case of eigenvectors of the highest *k* eigenvalues can be expressed as

(18) $$S_{K}=\sum_{m=1}^{k}\sum_{n=1}^{k}cos^{2}\left(\Theta_{mn}\right)$$

where $\Theta_{mn}$ is the angle between the *m*th eigenvector of A and the *n*th eigenvector of B.

## 3. Application to the Diagnosis of the Sensor and Actuator Faults of Vehicles

With the increasing complexity and widespread use of electrical and electronic components, functional safety is becoming of paramount concern in the automotive industry. Any faults that occur in these components can cause significant issues for vehicles, including degraded performance, increased noise and vibration, unintended torque requests, and more, all of which can compromise the functional safety of the vehicle. Therefore, it is of utmost importance to prioritize the development of reliable and resilient diagnosis and fault-tolerant control systems for electrified power trains. This is crucial to ensure the safe and reliable operation of vehicles on the road. Our work was motivated by the aforementioned challenges. Additionally, we aimed to produce results that are easily interpretable and reproducible. To achieve this, we demonstrated the application of the proposed monitoring procedure through simulations based on a vehicle dynamics model.

### 3.1. The Applied Model

In this research, a single-track dynamic non-linear vehicle model [51] is applied. It is used to describe the lateral motion of vehicles in the plane of the vehicle. It assumes that the left and right wheels are subject to an equal amount of lateral force, and it merges the four wheels of the vehicle on the two axles into two imaginary wheels, namely the front and rear wheels. It also assumes that the mass of the vehicle is concentrated on the gravitational center of the vehicle. This is the most basic vehicle model that can predict lateral motions. Due to its simpler representation, it facilitates analysis and requires much lower computational resources, making it suitable for real-time applications. The application of Newton’s second law for the motion along the *y* axis gives the lateral dynamics, and the moment balance about the *z* axis yields the yaw dynamics of the model. The dynamics are described using the following differential equations:

(19) $$\begin{array}{cc} ma_{y} & =F_{yf}+F_{yr}\\ I_{z}\ddot{\Psi } & =l_{f}F_{yf}-l_{r}F_{yr} \end{array}$$

After simplifications and transformations, the following equations can be described:

(20) $$\begin{array}{cc} \dot{X} & =v_{x}cos\left(\psi \right)-v_{y}sin\left(\psi \right)\\ \dot{Y} & =v_{x}sin\left(\psi \right)-v_{y}cos\left(\psi \right)\\ \dot{\psi } & =\frac{v_{x}}{l_{f}+l_{r}}tan\left(\delta \right)\\ m{\dot{v}}_{x} & =F_{x}+mv_{y}\dot{\psi }-2F_{yf}sin\left(\delta \right)-F_{a}-F_{r}\\ m{\dot{v}}_{y} & =-mv_{x}\dot{\psi }+2\left(F_{yf}cos\left(\delta \right)+F_{yr}\right)\\ I\ddot{\psi } & =2\left(l_{f}F_{yf}cos\left(\delta \right)-l_{r}F_{yr}\right) \end{array}$$

Here, *X* and *Y* are the longitudinal and lateral positional coordinates of the gravitational center of the vehicle. $v_{x}$ and $v_{y}$ are the longitudinal and lateral speeds, and ψ is the yaw of the vehicle. *m* and *I* denote the vehicle mass and yaw inertia, respectively. $F_{yf}$ and $F_{yr}$ denote the lateral tire forces at the front and rear wheels, which can be obtained using tire models [52]. $l_{f}$ and $l_{r}$ represent the distance from the center of the vehicle mass to the front and rear axles, and $F_{a}$ and $F_{r}$ are the air drag and rolling resistance.

The input of the system is the driving force $F_{x}$ and the steering angle δ. The measured outputs are the position coordinates *X* and *Y*, velocities $v_{x}$ and $v_{y}$, and yaw rate $\dot{\psi }$. Equation (21) summarizes the model variables:

(21) $$\begin{array}{cc} x & =[X,Y,\psi ,v_{x},v_{y},\dot{\psi }]\\ y & =[X,Y,v_{x},v_{y},\dot{\psi }]\\ u & =[F_{x},\delta ] \end{array}$$

### 3.2. Simulation Scenarios

This study deals with the detection and investigation of two sets of faults, similarly to reference [53].

- Actuator fault: This fault is in the steering angle command. The effective steering angle differs from the commanded steering angle by a constant 0.4 degrees.
- Sensor fault: The measured yaw rate does not correspond to the actual yaw rate, due to a positive 0.02 rad/s offset.

The states are simulated using the single-track model described in Section 3.1. During the simulation, the driving force $F_{x}$ was set to be constant and the steering angle δ had a sinusoidal input with a frequency of 0.05 and amplitude of 5°. For the measured variables (coordinates *x* and *y*, velocities $v_{x}$ and $v_{y}$, and yaw rate $\dot{\psi }$), white Gaussian noises were considered with covariances of

(22) $$\hat{R}=\left[\begin{array}{ccccc} 0.05 & 0 & 0 & 0 & 0\\ 0 & 0.05 & 0 & 0 & 0\\ 0 & 0 & 0.005 & 0 & 0\\ 0 & 0 & 0 & 0.005 & 0\\ 0 & 0 & 0 & 0 & 0.000005 \end{array}\right]$$

To simulate the sensor fault, 0.02 rad/s was added to the simulated yaw rate measurements starting from the 1000th time instant. The second scenario is an additive actuator fault, where the angle of the front wheel does not correspond to the commanded angle of steering. The same setting as in the sensor fault scenario was applied in the simulation. 0.4° was added to the sinusoidal steering angle input from the time instant 1000th.

**Figure 1:**
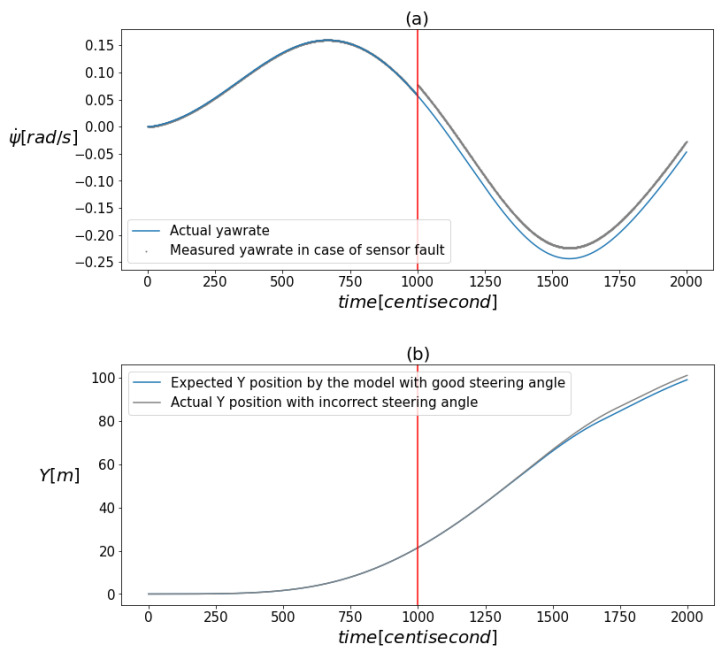
(**a**) demonstrates the deviation of the measured yaw rate from its actual value caused by the presence of a fault. (**b**) illustrates how the model’s prediction for the vertical position component differed from its actual value as a result of an incorrect steering input. The vertical red line indicates the moment when the error occurred.

Figure 1a,b visualize the effect of the faults. The figure illustrates that there was no significant shift in the values due to the faults when the measurements were plotted against time. This implies that the nature of the faults does not permit the use of conventional univariate signal-based fault diagnosis tools.

### 3.3. Applied Estimator Parameters

The particle filter estimation ran on the presented data, with the same model. The number of particles $N_{s}$ was set to 300 and the minimum threshold for effective sample size ϵ was set to 50% of all particles. The applied process and measurement noises were selected using a trial-and-error method, during which root mean squared error (RMSE) was used, and the guidelines of reference [54] were also followed. The selected parameters were

(23) $$R=\left[\begin{array}{ccccc} 0.1 & 0 & 0 & 0 & 0\\ 0 & 0.1 & 0 & 0 & 0\\ 0 & 0 & 0.001 & 0 & 0\\ 0 & 0 & 0 & 0.001 & 0\\ 0 & 0 & 0 & 0 & 0.000001 \end{array}\right]$$

(24) $$Q=\left[\begin{array}{cccccc} 0.1 & 0 & 0 & 0 & 0 & 0\\ 0 & 0.1 & 0 & 0 & 0 & 0\\ 0 & 0 & 0.001 & 0 & 0 & 0\\ 0 & 0 & 0 & 0.001 & 0 & 0\\ 0 & 0 & 0 & 0 & 0.001 & 0\\ 0 & 0 & 0 & 0 & 0 & 0.00001 \end{array}\right]$$

It is important to note that the Gaussian distribution with R covariance is not the same as the Gaussian distribution used to simulate measurement noise $\hat{R}$, although the two must be consistent with each other, because the latter has a strong impact on the best values to choose for the former.

### 3.4. Results and Discussion

**Figure 2:**
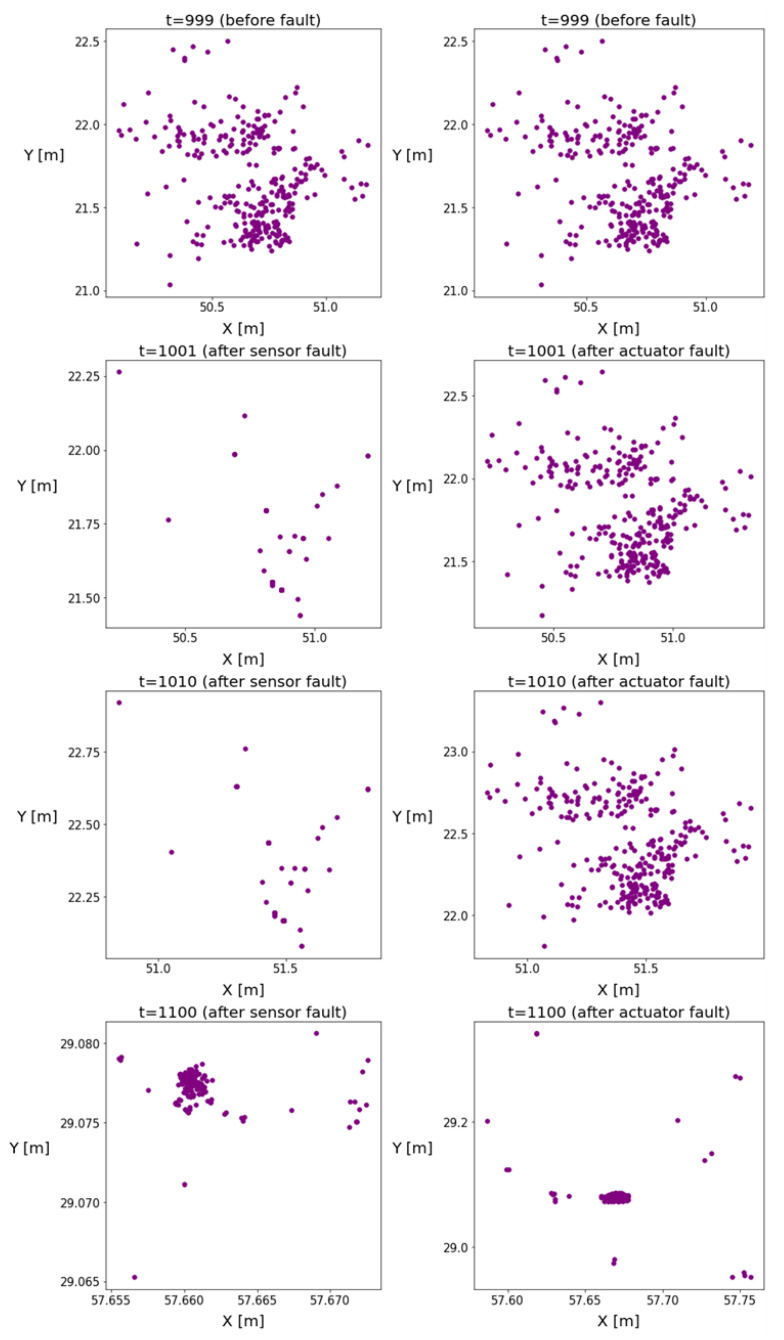
These figures illustrate the particle positions in the event of sensor or actuator faults. The left subplots show the evolution of particle positions in the case of sensor faults, while the right subplots show the evolution of particle positions in the case of actuator faults.

The position coordinates of the particles are plotted in Figure 2 to illustrate the changes in the homogeneity of the distribution. The figures indicate the emergence of a new operation mode with reduced variance, and the particles formed clusters over time, causing inhomogeneity in the distributions. The more intensive resampling reduced the number of particles. It can be observed that, in the event of an actuator fault, this phenomenon was delayed.

**Figure 3:**
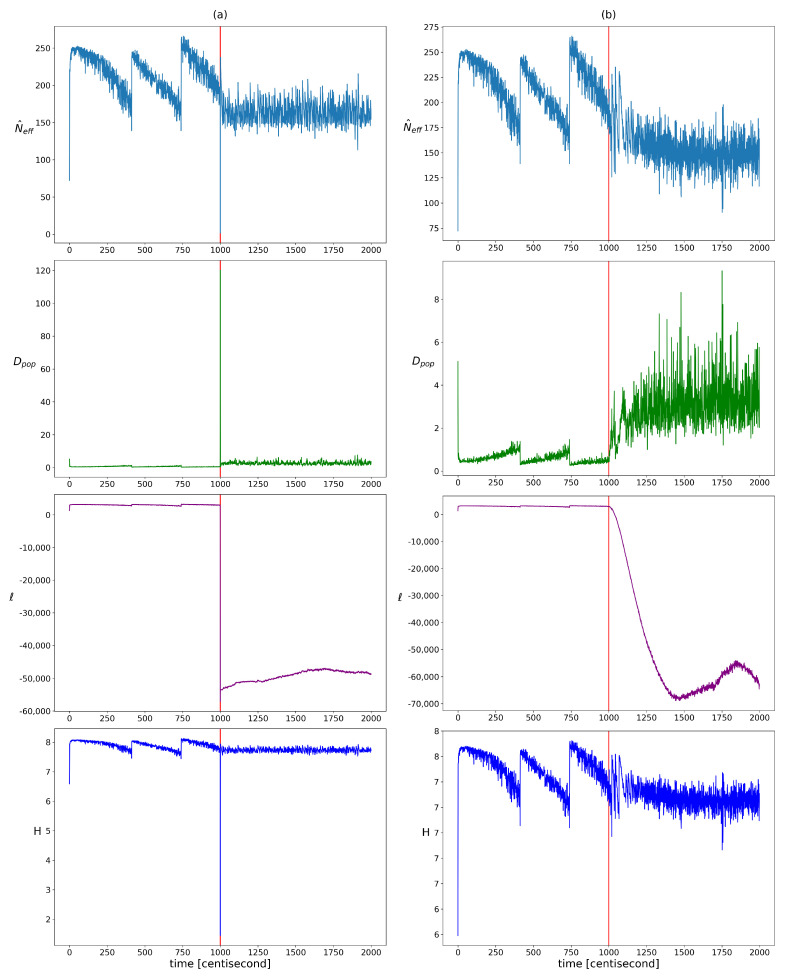
The changes in the different heterogeneity indicator values over time in cases of yaw rate sensor failure (figure (**a**)) and faulty steering angle (figure (**b**)). Listed from top to bottom: showing the effective sample size, the population diversity factor, the loglikelihood of particles, and the entropy of particle weights. The vertical red lines indicate the moment when the error occurred.

The change in homogeneity was examined using the qualitative indicators presented in Section 2.2.1. Their evolution over time is visualized in Figure 3. In the period before the error, a step-like pattern appeared for each indicator, which can be attributed to resampling. After the error, the resampling frequency increased, which broke this pattern. Based on the plots, the widely used log-likelihood function seemed to be the most meaningful indicator. In the presence of an error, its value decreased significantly, which allowed the choice of an absolute value threshold by expert judgment. In the case of the population diversity factor, the expected value clearly increased, but more frequent resampling results in higher variance, which can lead to false alarms. Hence, it is worth looking at its aggregated or expected value in a sliding window rather than its actual value. The use of entropy and effective sample size as diagnostic features requires more advanced methods. Both are heavily influenced by resampling, which keeps their values within a constant range. In order to define a reliable decision criterion, it is necessary to take into account the resampling-induced decreases, the variance changes, and the trend of their value. It can be seen that all the indicators change explosively in the event of an incorrect measurement and then quickly take on a constant value. This jump is not present in the case of an incorrect input, because the inconsistency between the model and the measurements develops gradually. It should be noted that, although not addressed in this work, it would be worthwhile to investigate the use of kurtosis, modality, and skewness measures to characterize the changes in the structure of the distribution (or possibly the use of clustering techniques, as the distributions are discrete). It would also be advisable to investigate the use of different types of distributions; for example, to investigate which of the different types of distribution best fits each type of error.

**Figure 4:**
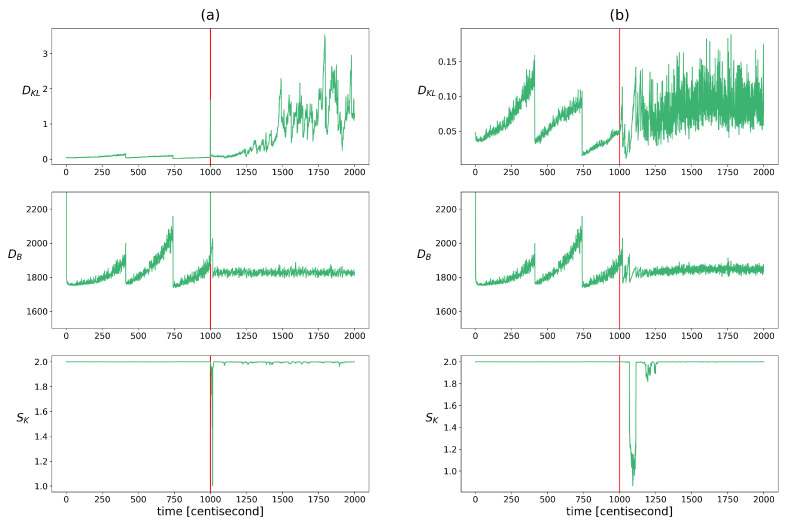
The changes in the indicators of update process monitoring over time in cases of yaw rate sensor failure (figure (**a**)) and faulty steering angle (figure (**b**)). Listed from top to bottom: Bhattacharyya distance between the prior and measurement densities, the KL divergence between the prior and posterior distributions, the Kranowsky similarity between the hyperplanes defined using the longest eigenvectors of the prior and posterior. The vertical red lines indicate the moment when the error occurred.

This inconsistency was checked using the indicators described in Section 2.2.3. The distance between the prior and the measurement density was measured using Bhattacharyya distance, the excess of the posterior information compared to the prior was quantified using KL divergence, and the similarity of their correlation pattern was analyzed with Krzanowsky similarity using the two largest eigenvectors of the priors and posteriors. Figure 4 shows the change in these values over time. The evolution of these indicators is very similar to that of the heterogeneity indicators. The KL divergence or relative entropy follows the same pattern as the entropy with the opposite sign. Therefore, its use as a fault diagnostic feature poses similar challenges. The pattern of the Bhattacharyya distances is similar to the pattern of the population diversity factors. It appears as a difference between the two fault types, where this distance increases over time to a greater extent in the case of a sensor fault. In the event of a measurement error, a momentary jump-like increase in the values can also be observed here. In the alignment of the hyperplanes defined by the two principal eigenvectors, a deviation appears after the appearance of the fault and the similarity value temporarily drops and then regains its maximum. The difference between the two types of failure is that this drop is delayed in the case of an erroneous input. Because of these characteristics, this measure can only be used to detect the occurrence of a fault, not its presence.

**Figure 5:**
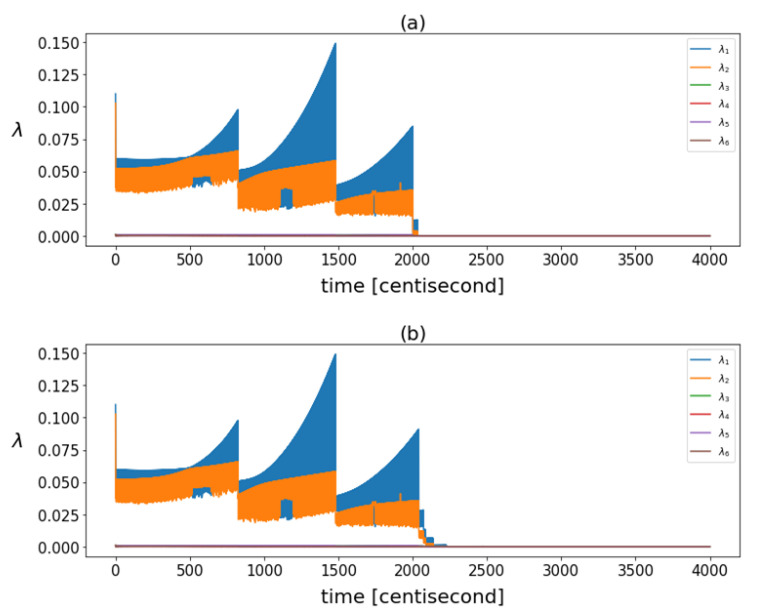
The eigenvalues calculated based on posterior covariance matrices in different time instants in case of (**a**) yaw rate sensor fault and (**b**) steering command fault.

The correlation structure was tracked over time. Figure 5 shows the eigenvalues of the posterior covariance matrices at different time instants. There was a significant decrease in these eigenvalues after failure, indicating a reduction in the overall variance of the data. While the filter was operating in steady-state mode, the variance of the particles increased continuously and fell back with each resampling.

**Figure 6:**
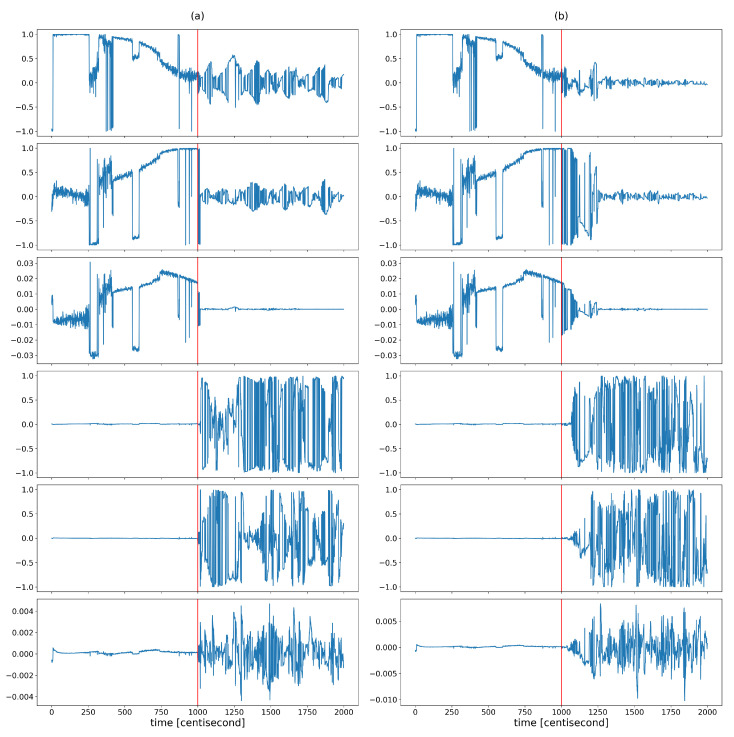
The different coordinates of the longest eigenvector computed from posterior covariances at different time instants in the case of (**a**) yaw rate sensor fault and (**b**) steering command fault. The vertical red lines indicate the moment when the error occurred.

**Figure 7:**
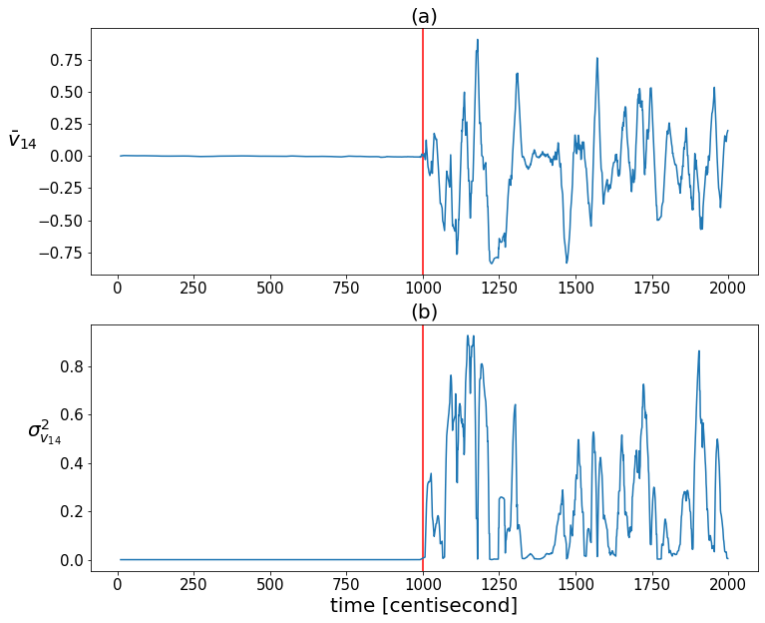
The mean values (figure (**a**)) and variances (figure (**b**)) of the fourth coordinates of the principal components of the posteriors in a sliding window of 20 data lengths. The vertical red lines indicate the moment when the error occurred.

The coordinates of the largest eigenvector $v_{1}^{\left(t\right)}=\left[v_{11}^{\left(t\right)},v_{12}^{\left(t\right)},...,v_{16}^{\left(t\right)}\right]$ computed from the posterior covariances are monitored over time to capture the shaping of the correlation structure (Figure 6). The figure shows that the variability of the correlation structure changed significantly, after the fault occurrence. The values of the first three coordinates were set close to zero. These three coordinates correspond to the contributions of the first three state variables (which are the zeroth order variables) to the principal component, represented by the eigenvector with the largest eigenvalue. The values of the other three coordinates, corresponding to the contributions of the first-order derivative variables to the principal component, began to oscillate to a great extent as a result of the error. During this oscillation, they had absolute values close to one. This shows that as a result of the error, the correlation between the lower-order variables decreased and the higher-order variables tended to determine the correlation structure. The absolute values close to one indicate that the variance of the state distribution at a given moment can always be related to a specific higher-order state variable to a great extent. As these coordinates alternated between −1 and 1, no stable value was formed based on which the type of error could be distinguished. All this shows that the correlation structure is shaped by random fluctuations in the probability distribution of the rate of change of the system, rather than by a specific effect characteristic of a unique fault, so that the information obtained from the correlation pattern cannot be used for fault identification. However, the variability characteristic of the correlation structure is promising for detection purposes, as the statistical properties of these coordinates changed sharply as a result of the failure. Figure 7 illustrates this well by plotting the means and variances of the fourth coordinates of the principal component (this coordinate is related to the velocity component $v_{x}$) in a sliding window of 20 data lengths in the case of the sensor fault scenario. Both statistical properties changed greatly in the presence of error.

**Figure 8:**
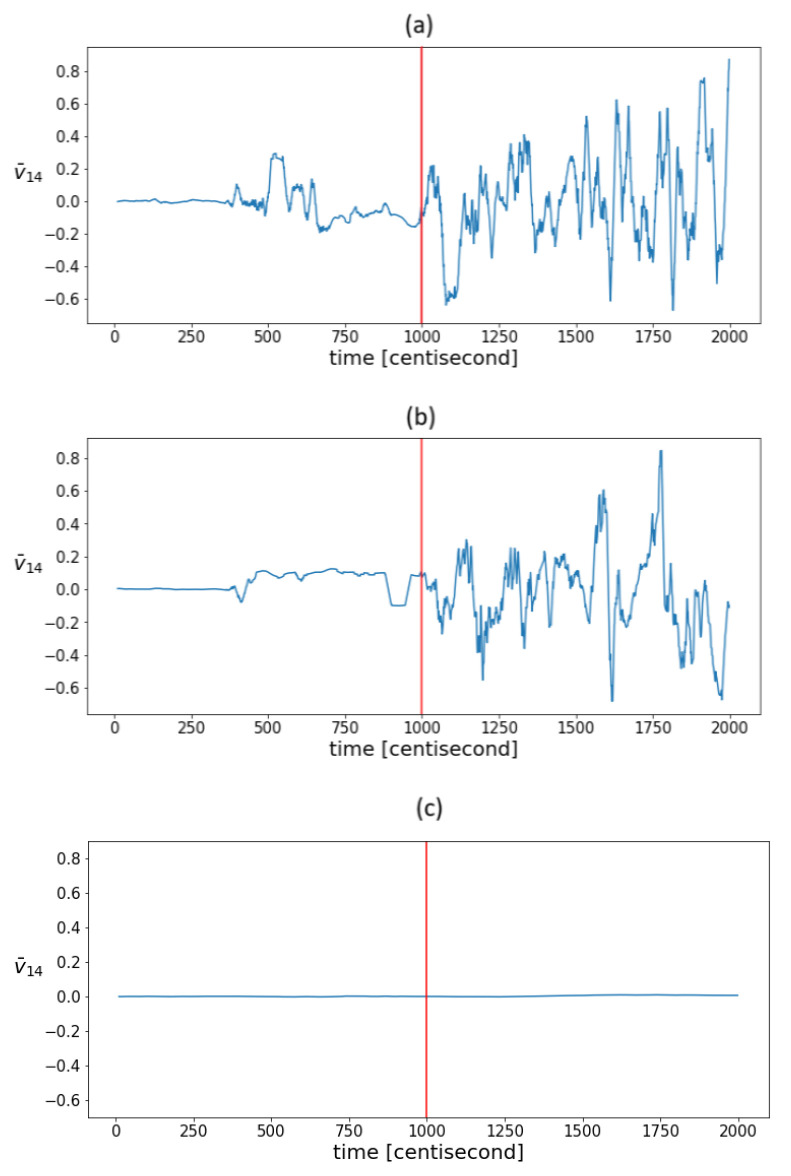
The evolution of mean values in a sliding window of the fourth coordinates of the principal components in the case of estimations with (**a**) an order of magnitude higher measurement noise, (**b**) an order of magnitude higher process covariance Q, and (**c**) an order of magnitude higher output covariance R. The vertical red lines indicate the moment when the error occurred.

It is to be expected that as measurement noise increases, so will statistical fluctuations, even under normal operation conditions. To test this, state estimation was also performed in the presence of simulated measurement noise that was an order of magnitude higher. Figure 8a shows the mean value of the fourth coordinate of the principal component in the sliding window in the case of an order of magnitude higher measurement noise. It can be seen that the efficiency of the indicator decreased, but not significantly, as the same pattern still appeared. The sensitivity of the indicators to measurement noise for a given model and filter parameters is determined by the capabilities of the PF. Thus, the noise should be considered primarily through the tuning of the PF algorithm. It can also be assumed that by increasing the freedom of the particles, the statistical variance will increase, and the efficiency of the method will be compromised. To test this, we performed state estimation with process covariance values that were an order of magnitude higher. With such values, the deviation of the estimate itself proved to be unacceptable; however, the same variability pattern still appeared in the correlations (Figure 8b). By increasing the measurement density, the degree of fluctuations can be reduced. In this case, the conclusion of the model became more and more decisive compared to the measurement during estimation. As a result of which, the correlation structure remained intact even under the influence of the fault, which on the one hand was at the expense of detectability, and on the other hand, the correlation structure still did not indicate the nature of fault-relevant information. To test this assumption, the estimation was performed using output density covariance values that were one order of magnitude higher. This assumption was confirmed. Figure 8c shows the evolution of the mean values of the fourth eigenvector coordinate. The graph shows that the value remained close to zero throughout the process. In summary, in the range where state estimation works, correlation variability indicators also work, and hence, they can aid the tuning of particle filter-based fault diagnostics algorithms.

## 4. Conclusions

The objective of this study was to gain insight into the operation of a particle filter used to investigate what type of information and features can be extracted for fault detection and identification purposes. A monitoring procedure was proposed to track the correlation and information structure of discrete probability distributions estimated using a particle filter operated with a normal operation model, to extract fault-specific information. The proposed method evaluated the heterogeneity of the posterior distribution particles, their correlation, and the consistency of the prediction and observations of the model. It was highlighted that the log-likelihood-based indicator was effective for fault detection. The indicators developed changed significantly in the case of faults, and sensor errors caused abrupt changes that were different from actuator errors. The proposed correlation structure analysis revealed shifts in state variance due to faults and helped to tune particle-filter-based fault diagnostic algorithms. Future research should focus on the development of analytical methods that compare distribution differences to fault detection accuracy and corresponding measures, which could allow for targeted tuning. It would also be worth considering the application of hybrid system models that estimate discrete modes and allow complex varying dynamics to be captured, as well as the application of clustering techniques to explore different operating and fault modes.

## Data Availability

Data are contained within the article.
